# Volume XXIX

**Published:** 1889-08

**Authors:** 


### *VOLUME XXIX.*

WITH the present number, the JOURNAL begins its twenty-ninth volume in the new series. It will be observed that we have made some changes in its make-up that will result in affording more reading matter, as well as in improving the general appearance of the magazine. The additions to our staff of Associate Editors cannot fail to prove of advantage, and is the immediate result of the combination with the *Medical Press*, that has heretofore been announced. Though we part with Drs. Angell and Crego, to whom the JOURNAL is indebted for efficient work, we expect our readers will hear from them as frequently as their busy lives will permit.

Without boastful promises, we yet enter upon the work of preparing the new volume with high hopes for its successful career; to which end we invoke the support and sympathy of the medical profession of this immediate vicinity, and of our readers, wherever resident throughout the world.

